# Acute effects of different exercise forms on executive function and the mechanism of cerebral hemodynamics in hospitalized T2DM patients: a within-subject study

**DOI:** 10.3389/fpubh.2023.1165892

**Published:** 2023-05-24

**Authors:** Haolin Wang, Wei Tang, Yanan Zhao

**Affiliations:** ^1^School of Sports Science and Physical Education, Nanjing Normal University, Nanjing, China; ^2^Department of Endocrinology, Geriatric Hospital of Nanjing Medical University, Nanjing, China

**Keywords:** aerobic exercise, resistance exercise, type 2 diabetes, integrated concurrent exercise, executive function, brain activation

## Abstract

**Objective:**

This study aimed to investigate the acute effects of aerobic exercise (AE), resistance exercise (RE), and integrated concurrent exercise (ICE; i.e., AE plus RE) on executive function among hospitalized type 2 diabetes mellitus (T2DM) inpatients, and the mechanism of cerebral hemodynamics.

**Methods:**

A within-subject design was applied in 30 hospitalized patients with T2DM aged between 45 and 70 years in the Jiangsu Geriatric Hospital, China. The participants were asked to take AE, RE, and ICE for 3 days at 48-h intervals. Three executive function (EF) tests, namely, Stroop, More-odd shifting, and 2-back tests, were applied at baseline and after each exercise. The functional near-infrared spectroscopy brain function imaging system was used to collect cerebral hemodynamic data. The one-way repeated measurement ANOVA was used to explore training effects on each test indicator.

**Results:**

Compared with the baseline data, the EF indicators have been improved after both ICE and RE (*p* < 0.05). Compared with the AE group, the ICE and RE groups have demonstrated significant improvements in inhibition (ICE: MD = − 162.92 ms; RE: MD = −106.86 ms) and conversion functions (ICE: MD = −111.79 ms; RE: MD = −86.95 ms). Based on the cerebral hemodynamic data, the beta values of brain activation in executive function related brain regions increased after three kinds of exercise, the EF improvements after the ICE showed synchronous activation of blood flow in the dorsolateral prefrontal cortex (DLPFC), the frontal polar (FPA) and orbitofrontal cortex (OFC), the improvement of inhibitory function after RE displayed synchronous activation of DLPFC and FPA, and AE mainly activates DLPFC. The HbO_2_ concentration in the pars triangularis Broca's area increased significantly after AE, but the EF did not improve significantly.

**Conclusion:**

The ICE is preferred for the improvements of executive function in T2DM patients, while AE is more conducive to the improvements of refresh function. Moreover, a synergistic mechanism exists between cognitive function and blood flow activation in specific brain regions.

## 1. Introduction

Diabetes mellitus is the third non-communicable disease threatening human health worldwide (1). According to the global diabetes map published by the International Diabetes Federation, 537 million people were living with diabetes worldwide in 2021, and the number is expected to rise to 643 million by 2030 and 783 million by 2045. The number of diabetics in China accounts for one-quarter of the world (~140.8 million), ranking first globally, and is expected to increase to 175 million by 2045 (2).

As the highest and most complex cognitive function (3), executive functions (EF) include three sub-functions: inhibition, refresh, and transformation. Clinical evidence showed that the inhibitory, conversion, and refresh function scores in T2DM patients were 12, 21, and 7% lower than those age-matched non-T2DM patients, respectively (4), and the risk of EF injury in middle-aged and older T2DM patients was 19% higher than that in age-matched healthy people (5). Notably, T2DM inpatients had a higher risk of cognitive impairment due to decreased physical activity, depressed mood, and social isolation than patients with T2DM in the community or outpatient setting (6). Studies have found that 13–63% of hospitalized patients have cognitive impairment of varying degrees (7). The incidence of cognitive impairment in hospitalized patients with T2DM was 37.9%, 50.7% of which were EF injuries (8).

As an economical, green, and safe way to prevent diabetes, exercise has been applied to improve EF in hospitalized patients with T2DM (9). To date, aerobic exercise (AE) and resistance exercise (RE) are the two main exercise formats in T2DM patients, and integrated concurrent exercise (ICE; i.e., AE plus RE) has got increased recognition in its efficacy in cognitive function among T2DM patients (10, 11). However, no consensus has been made regarding the preferred exercise format in EF improvement among T2DM inpatients. Some evidence even revealed little effects of 24 weeks of balance combined with resistance training (12) or 10 years of AE (13) on EF in patients with T2DM. Except for the differences in test parameters, the underlying mechanisms of the inconsistent results toward exercise efficacy in EF of T2DM patients remains unknown.

Functional near-infrared spectroscopy (fNIRS) is a non-invasive and real-time way to monitor the cerebral blood oxygen by taking the brain tissue blood volume and oxygen as an information carrier. Cerebral hemodynamics refers to the changes and distribution of blood volume and oxygen in the cerebral cortex. Studies applying fNIRS and EF tasks together have revealed that an acute AE can improve EF and increase cerebral blood flow activation levels among older adults and college students (14–16). In addition, one study has applied the same exercise in both healthy and type 1 diabetic adolescents, and the results showed that people with diabetes would have higher activation in the frontal-parietal network. However, few studies have examined the exercise effects on EF and cerebral blood flow activation together among T2DM inpatients.

Based on the current knowledge and study problems, this study aimed to explore the acute effects of AE, RE, and ICE on each EF indicator in inpatients with T2DM, and the fNIRS was used to explore the underlying cerebral hemodynamic mechanism.

## 2. Methods

### 2.1. Study design and participants

A cross-sectional, within-subject design was applied in this study. The sample size was estimated using the G^*^power 3.1 software. Based upon the results from a related study which examined the acute effects of AE on EF in older adults (16), a moderate effect (*f* = 0.27) was applied with 80% power to calculate the required sample size. Considering 10% drop-out rate, 21 participants were needed. A total of 30 hospitalized T2DM patients aged between 45 and 70 years were recruited from the Endocrinology Department of the Jiangsu Geriatric Hospital. Participant recruitment was done by a chief physician and a researcher. Eligible participants should be (1) with no severe diabetic retinopathy, diabetic nephropathy, or diabetic foot; (2) with normal vision and color discrimination, no color weakness, color blindness, or serious eye disease; and (3) stable and can complete three (non-continuous) sessions of moderate-intensity AE or RE under special supervision for 20 min. The exclusion criteria were (1) glycosylated hemoglobin (HbA1c) ≥ 9%; (2) cognitive dysfunction (Mini-mental State Examination score: education level of primary school < 20 and education level of junior high school and above < 24); (3) suffering from depression, mental illness, or a family history of mental illness; (4) drug and alcohol abuse (daily taking more than three drugs); and (5) exercise limitations, such as muscle insufficiency, joint disease, cardiovascular disease, respiratory disorders, or other exercise contraindications to T2DM. Participants were required to sign informed consent before the formal study but were blinded for the primary test outcome of the study.

According to the ACSM's classification of AE intensity (17) and the commonly used exercise intensity for diabetic participants (18, 19), the moderate intensity of AE was set as 60–70% of the maximum heart rate of each individual (220—age). At the same time, based on the ACSM's recommendations on exercise intensity for patients with no regular exercise habits (20) and with the consideration of the commonly applied exercise intensity (21), the moderate intensity of RE was set at 60–70% 1RM. The participants were asked to perform 30 min of AE, RE, and ICE, including 20 min exercise and 10 min warm-up and stretching before and after exercise on separate days with 48 h time intervals. All the participants finished the four times tests in a four-part sequence using a Latin square counterbalancing design to reduce the impact of potential practice effects (22). The assignment of the subjects' exercise order was done by the researcher alone. To avoid the potential time effects on blood glucose fluctuation, tests were conducted at 9:30–10:30 a.m. This study has obtained the approval of the ethics committee of Nanjing Normal University (2022060013).

### 2.2. Experimental procedure

Before the experiment, basic information about the participants was collected, and cognitive function, cardiopulmonary function, and muscle strength were evaluated to determine the feasible range of moderate-intensity exercise. Four kinds of strength training equipment (chest push/row trainer, KY-701; inner and lateral thigh muscle trainer, KY-702; kick hook trainer, KY-703; and abdominal muscle and back muscle trainer, KY-705) were used to evaluate the 1RM value of individuals by counting the number and resistance level of specific movements completed by corresponding muscle groups within 1 min.

The formal experimental procedure of this study consists of four parts: baseline tests, AE, RE, and ICE. Participants are suggested to be free of vigorous exercise 12 h before tests and arrive at the lab 15 min before formal tests. AE was performed using a Magneto bicycle (Zhiqi, HG-HRUB150T). A heart rate belt (Polar, OH1) was used to monitor heart rate during exercise, and the average intensity was maintained within 60–70% of the maximum heart rate. RE is completed based on the four kinds of strength training equipment, which were adjusted to ensure that the individual exercise intensity was within the 60–70% 1RM. Each movement should be completed 8–12 times/group×3 groups. The 20 min ICE included AE and RE, and 10 min for each.

After each test session, the self perceived exertion was assessed to ensure the whole exercise was performed at the moderate intensity level, and participants' blood glucose was measured before and immediately after exercise to ensure exercise safety, we paid close attention to the levels of various glucose and lipid metabolism indexes of patients during the exercise experiment.

### 2.3. Outcomes

Three EF tests were performed using the E-Prime 3.0 system, including the Stroop test, the More-odd shift test, and the 2-back test. The system records the participants' reaction time and accuracy during the test. In addition, we further extracted the Stroop interference (the difference between the mean value of the response time of the inconsistent task and the mean value of the response time of the consistent task), the switch response time of the conversion task (the difference between the mean value of the response time of the converted part and the mean value of the response time of the non-converted part), and the correct response time and the number of the correct response of the refresh task. Referring to existing studies (23, 24) and fNIRS data collection in this experiment and the actual situation of the participants, the specific settings of the three executive function tests are as follows.

In each Stroop trial, the computer screen presented a 500-ms “+”, a 500-ms color word stimulus, and a 3,000-ms empty screen rest in the middle. Color word stimulus refers to four kinds of Chinese characters randomly matched with red, blue, green, and yellow font colors (divided into two types of stimulus, consistent and inconsistent, according to whether the meaning and color are consistent). Participants are asked to press the keyboard to judge the color of the stimulus as soon as possible (red according to D, yellow according to F, green according to H, and blue according to J). The tests included 10 trials for practice and 62 trials for formal trials (16 trials for consistent stimulation and 46 trials for inconsistent stimulation) for a total of 4.8 min. In each trial of the More-odd shifting test, a 500 ms fixation point “+” was presented in the center of the computer screen, followed by 1,500 ms of digital stimulation (1–9, excluding 5) and 3,000 ms of empty screen rest. The participants were required to convert and judge according to the color of the numbers. When the numbers were black, the size of the numbers was judged (according to the F for <5 and according to the L for more than 5), while the number is green means the requirements to judge the parity (according to the J for odd numbers and according to the K for even numbers). This task included 18 trials for practice and 88 trials for formal tests. The duration is 8.83 min. In each trial of the 2-Back test, the computer screen presented 1,000 ms of stimulus numbers (including 2, 4, 5, 7, 9) in the center, followed by a 3,000-ms empty screen rest. The participants were asked to judge the consistency of numbers by checking if the number was the same as the number presented separately (according to the Y for same and according to the N for different). The task consisted of practicing 12 trials and formally testing 54 trials with a duration of 4.4 min.

The multi-channel fNIRS system (NirSmart-6000A, Danyang Huichuang Medical Equipment Co., Ltd., China) was used to continuously collect the change data of local cerebral oxygenated hemoglobin concentration (HbO_2_) during EF tests. According to the distribution of EF neural-activated brain regions, the fNIRS optical cap mainly covered the prefrontal cortex (PFC) in this experiment. The cap is designed based on the 10/20 international standard lead system. It consists of seven light source transmitting probes and seven light source receiving probes to form 19 effective channels. The light source wavelength is 730 nm, the receiving wavelength is 850 nm, the sampling rate is 11 Hz, and the average distance between the emitter and the detector is 30 mm.

## 3. Data collection

The E-Data Aid of the E-prime 3.0 system was used to derive the accuracy and overall response time data of EF tests in four experiments per participant, and then we calculated the mean of the data. According to the mark set in the E-prime 3.0 system, the fNIRS optical density data of each trial in the test were intercepted and preprocessed using the Preprocess module in NirSpark1.7.5. The signal standard deviation threshold was set as 6 and the peak threshold as 0.5. The spline interpolation method was used to identify and remove motion artifacts. The noise and interference signals were filtered at 0.01–0.2 Hz. According to the modified Beer–Lambert law, optical density was converted to blood oxygen concentration. The beta value of brain activation after different exercises was calculated in the general linear model (GLM) module of NirSpark1.7.5, and the beta value was used as an indicator to measure the activation degree of corresponding brain regions. The descriptive statistics were reported as mean ± standard deviation.

## 4. Data analysis

The accuracy and response time of each EF test of 30 participants and the mean value of HbO_2_ concentrations in 19 channels were analyzed by using SPSS25.0 statistical software for one-way repeated measures ANOVA for different exercise types (Baseline, AE, RE, and ICE). The Bofferoni was used to correct the significance level of multiple comparisons in the *post-hoc* analysis. An independent sample *t*-test was used to examine gender effects on demographic indicators. The *p*-value of < 0.05 was considered a statistical significance.

## 5. Results

### 5.1. Basic information of participants

A total of 36 qualified participants were recruited for this study, of which 30 completed the whole experiment, six dropped out during the experiment, three did not complete three times of exercise due to illness limitation, one did not complete three times of refresh function tests due to physical discomfort after exercise, and one was discharged early due to conflict between temporary treatment arrangement and experiment time. Few female participants (1/5 of all participants) were affected by exercise willingness and illness. There were significant differences between male and female participants only in height (*t* = 4.58, *p* < 0.001). There were no significant gender differences in other indicators (*p* > 0.05). In addition, the mean BMI of women was higher than that of men, which was in the overweight range (≥24 kg/m^2^). The average WHR of the participants was generally high, indicating abdominal obesity. Participants were in the age range of 45–70 years, and the median age was 65 years. Approximately 93.3% of the participants had a high school education or above and were well educated without cognitive impairment (Table 1). All participants had their conditions under control during hospitalization and obtained the doctor's permission to exercise before carrying out the exercise experiment. There were no aggravations or injuries during the whole experiment.

**Table 1:** Basic information of participants.

**Index**	***N*** **(%)/Mean (**±**SD)**		
	**Male**	**Female**	**Overall**
*N* ^a^	24(80%)	6(20%)	30
*N* ^b^	22 (92%)	6 (100%)	28 (93%)
Age (years)	64.71 ± 5.47	62.00 ± 8.00	64.17 ± 5.99
Height (cm)	171.83 ± 5.50	160.17 ± 5.95^*^	169.50 ± 7.25
Weight (kg)	69.55 ± 9.64	65.40 ± 8.75	68.72 ± 9.47
BMI (kg/m^2^)	23.48 ± 2.95	25.45 ± 3.15	23.87 ± 3.05
WHR	0.93 ± 0.03	0.94 ± 0.03	0.93 ± 0.03
MMSE Score	27.05 ± 1.62	27.00 ± 2.37	26.77 ± 2.05
FBG (mmol/L)	7.50 ± 2.12	7.26 ± 2.30	7.45 ± 2.11
2hPG (mmol/L)	14.74 ± 4.10	14.42 ± 3.34	14.68 ± 3.92
HbAc1 (%)	8.56 ± 2.31	7.38 ± 1.19	8.32 ± 2.17
TC (mmol/L)	4.10 ± 1.12	4.23 ± 0.96	4.13 ± 1.07
TG (mmol/L)	1.61 ± 1.07	1.31 ± 0.39	1.55 ± 0.97
LDL-C (mmol/L)	2.39 ± 0.96	2.41 ± 0.67	2.39 ± 0.89
HDL-C (mmol/L)	0.96 ± 0.25	1.07 ± 0.10	0.98 ± 0.23

* *p* < 0.05.

*N*^a^, number of all participants; *N*^b^, number of participants with middle school experience and above; BMI, body mass index; WHR, waist-to-hip ratio; MMSE, Mini-mental State Examination; HbA1c, glycosylated hemoglobin type A1c; TC, total cholesterol; TG, triglyceride; FBG, fasting blood glucose; 2hPG, 2 h postprandial blood glucose; LDL-C, low-density lipoprotein; HDL-C, high-density lipoprotein.

### 5.2. Executive function data

#### 5.2.1. Inhibition function data

The results of one-way repeated measurement analysis of variance showed that the four tests had a significant effect on the accuracy of inconsistent tasks [*F*_(3,87)_ = 5.88, *p* = 0.003, partial η^2^ = 0.169]. Compared with the baseline level, RE and ICE can effectively improve the accuracy of inconsistent tasks (resistance: mean difference = 6.40%, *p* = 0.04; ICE: mean difference = 8.19%, *p* = 0.012). After RE, the accuracy of consistent tasks improved the most (RE> ICE> AE); and after ICE, the accuracy of inconsistent tasks improved the most (ICE> RE> AE).

All four tests showed significant effects on response time under consistent [F_(3,87)_ = 14.38, *p* < 0.001, partial η^2^ = 0.331] and inconsistent [F_(3,87)_ = 12.47, *p* < 0.001, partial η^2^ = 0.301] tasks. Both RE and ICE significantly reduced reaction time for consistent (RE: mean difference = −180.09 ms, *p* < 0.001; ICE: mean difference = −219.39 ms, *p* < 0.001) and inconsistent (RE: mean difference = −149.54 ms, *p* = 0.01; ICE: mean difference = −205.59 ms, *p* < 0.001) tasks compared to baseline levels. In addition, compared with AE, both consistent (ICE: mean difference = −129.12 ms, *p* = 0.002; RE: mean difference = −89.83 ms, *p* = 0.006) and inconsistent (CE: mean difference = −162.92 ms, *p* < 0.001; RE: mean difference = −106.86 ms, *p* = 0.001) task response times were significantly reduced after ICE and RE.

There was no significant difference in Stroop interference [*F*_(3,87)_ = 1.13, *p* = 0.33, partial η^2^ = 0.038] to the four tests, but the improvement trend of the amount of reaction time conflict after the three types of exercise was from large to small: ICE > RE> AE.

#### 5.2.2. Conversion function data

The results of one-way repeated measurement analysis of variance showed that there were significant differences in the accuracy [*F*_(3,87)_ = 3.80, *p* = 0.013, partial η^2^ = 0.116] and response time [*F*_(3,87)_ = 0.51, *p* < 0.001, partial η^2^ = 0.249] of the four tests. Compared with the baseline level, the task accuracy (mean difference = 7.68%, *p* < 0.001) and response time (mean difference = −100.69 ms, *p* < 0.001) were significantly improved after ICE. After RE, only the task response time improved significantly (mean difference = −75.85 ms, *p* = 0.039). In addition, compared with AE, ICE (mean difference = −111.79 ms, *p* < 0.001) and RE (mean difference = −86.95 ms, *p* = 0.021) showed a significant reduction in task response time, but no significant difference in switch reaction time. However, compared with the baseline level, the conversion response showed a decreasing trend after the three types of exercise, and the decreasing amplitude in descending order was ICE > RE > AE.

#### 5.2.3. Refreshing function data

The four tests showed significant differences in the number and rate of correct responses [*F*_(3,87)_ = 3.54, *p* = 0.018, partial η^2^ = 0.109], reaction time [*F*_(3,87)_ = 6.33, *p* = 0.001, partial η^2^ = 0.179], and correct response time [*F*_(3,87)_ = 6.11, *p* = 0.004, partial η^2^ = 0.174]. Compared with the baseline level, the number of correct responses (mean difference = 6.60, *p* = 0.027), correct rate (mean difference = 14.99%, *p* = 0.027), and reaction time (mean difference = −141.62 ms, *p* = 0.007) were significantly improved after ICE. Reaction time (mean difference = −142.97 ms, *p* = 0.002) and correct response time (mean difference = −157.06 ms, *p* = 0.007) decreased significantly after RE, while AE only improved in response time to correct answer (mean difference = −207.38 ms, *p* = 0.046). In addition, there was no significant difference between the three different types of exercise in the performance of refresh function tasks, but all the test indicators showed a certain degree of improvement trend; the correct rate and the number of correct responses increased the most after ICE, the response improved the most after RE, and the correct response decreased the most after AE (Table 2).

**Table 2:** Performance of EF tests after different exercises.

**EF**	**Group**		**Baseline**	**AE**	**RE**	**ICE**
Inhibitory function	Accuracy rate (%)	Consistent	92.91 ± 1.16	92.29 ± 1.36	97.29 ± 0.54	92.65 ± 1.87
		Inconsistency	83.44 ± 1.93	86.34 ± 1.64	89.84 ± 1.38^#^	91.63 ± 1.14^#^^*^
	Overall reaction time (ms)	Consistent	1,166.83 ± 304.72	1,076.57 ± 262.52	986.74 ± 252.88^#^^*^	947.45 ± 200.08^#^^*^
		Inconsistency	1,285.08 ± 291.78	1,242.40 ± 281.87	1,135.54 ± 270.43^#^^*^	1,079.49 ± 253.73^#^^*^
	Stroop interference (ms)	Inconsistency-Consistent	118.25 ± 123.79	165.83 ± 118.53	148.80 ± 86.91	132.05 ± 142.15
Conversion function	Accuracy rate (%)		85.08 ± 1.39	87.66 ± 1.32	89.77 ± 1.13	92.76 ± 0.83^#^
	Overall reaction time (ms)		1,124.08 ± 167.07	1,135.17 ± 186.18	1,048.22 ± 165.44^#^^*^	1,023.39 ± 185.37^#^^*^
	Switch reaction time (ms)		382.42 ± 162.94	365.72 ± 205.07	365.17 ± 148.90	341.98 ± 145.11
Refresh function	Accuracy rate (%)		66.68 ± 2.44	75.13 ± 2.02	77.77 ± 2.12	81.67 ± 1.77^#^
	Correct responses (n)		29.34 ± 10.75	33.06 ± 8.88	34.22 ± 9.34	35.93 ± 7.80^#^
	Correct reaction time (ms)		1,120.25 ± 296.26	912.87 ± 190.57^#^	963.19 ± 209.97^#^	968.34 ± 225.17^#^
	Reaction time (ms)		1,107.72 ± 265.55	1,027.23 ± 216.36	964.75 ± 210.00^#^	966.10 ± 219.57^#^

AE, aerobic exercise; RE, resistance exercise; ICE, Integrated concurrent exercise.

# *p* < 0.05, indicating a significant difference from the baseline level.

* *p* < 0.05, indicating significant difference with aerobic exercise.

### 5.3. Cerebral hemodynamics data

#### 5.3.1. Brain activation during functional inhibition tests

One-way repeated measure analysis of variance showed that under inconsistent tasks, the four tests showed significant differences in dorsolateral prefrontal cortex (DLPFC; Channel S3-D3: F_(3,87)_ = 3.856, *p* = 0.012, partial η^2^= 0.117; S5-D5: F_(3,87)_ = 4.694, *p* = 0.012, partial η^2^ = 0.139; S7-D3: F_(3,87)_ = 3.456, *p* = 0.020, partial η^2^ = 0.106), orbitofrontal area (OFC; S2-D1: F_(3,87)_ = 8.350, *p* < 0.001, partial η^2^ = 0.224) and inferior prefrontal gyrus (IPG; S4-D3; F_(3,87)_ = 3.033, *p* = 0.033, partial η^2^ = 0.095). Under the consistent task, there were significant differences in DLFPC (S5-D5: F_(3,87)_ = 4.314, *p* = 0.007, partial η^2^ = 0.129) and frontal polar region (FPA; S6-D5: F_(3,87)_ = 3.262, *p* = 0.025, partial η^2^ = 0.101) in the four tests.

**Figure 1:**
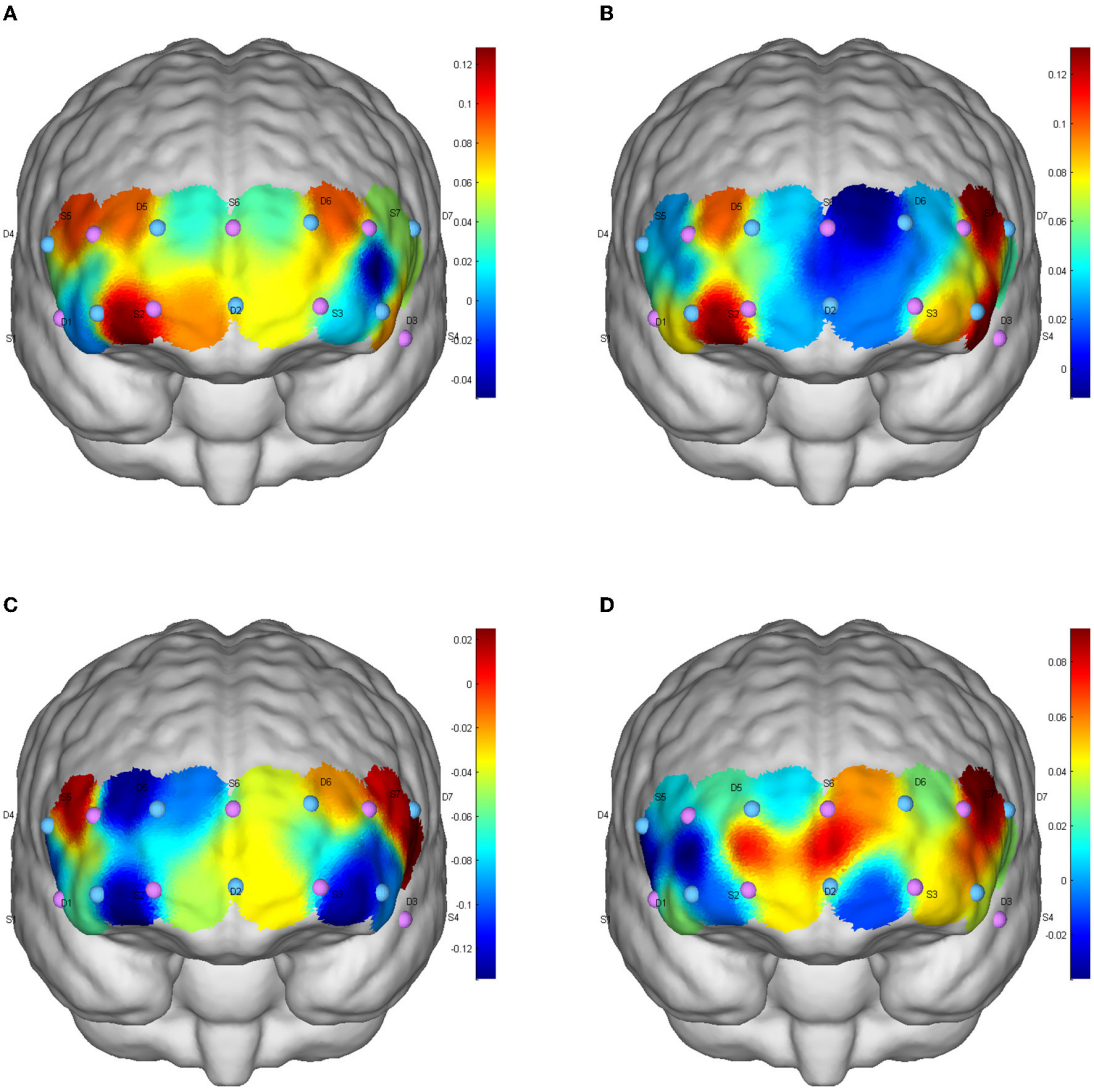
Inhibition function of CBF activation after different exercises in inconsistent tasks. **(A)** Baseline level; **(B)** aerobic exercise; **(C)** resistance exercise; **(D)** integrated concurrent exercise.

**Figure 2:**
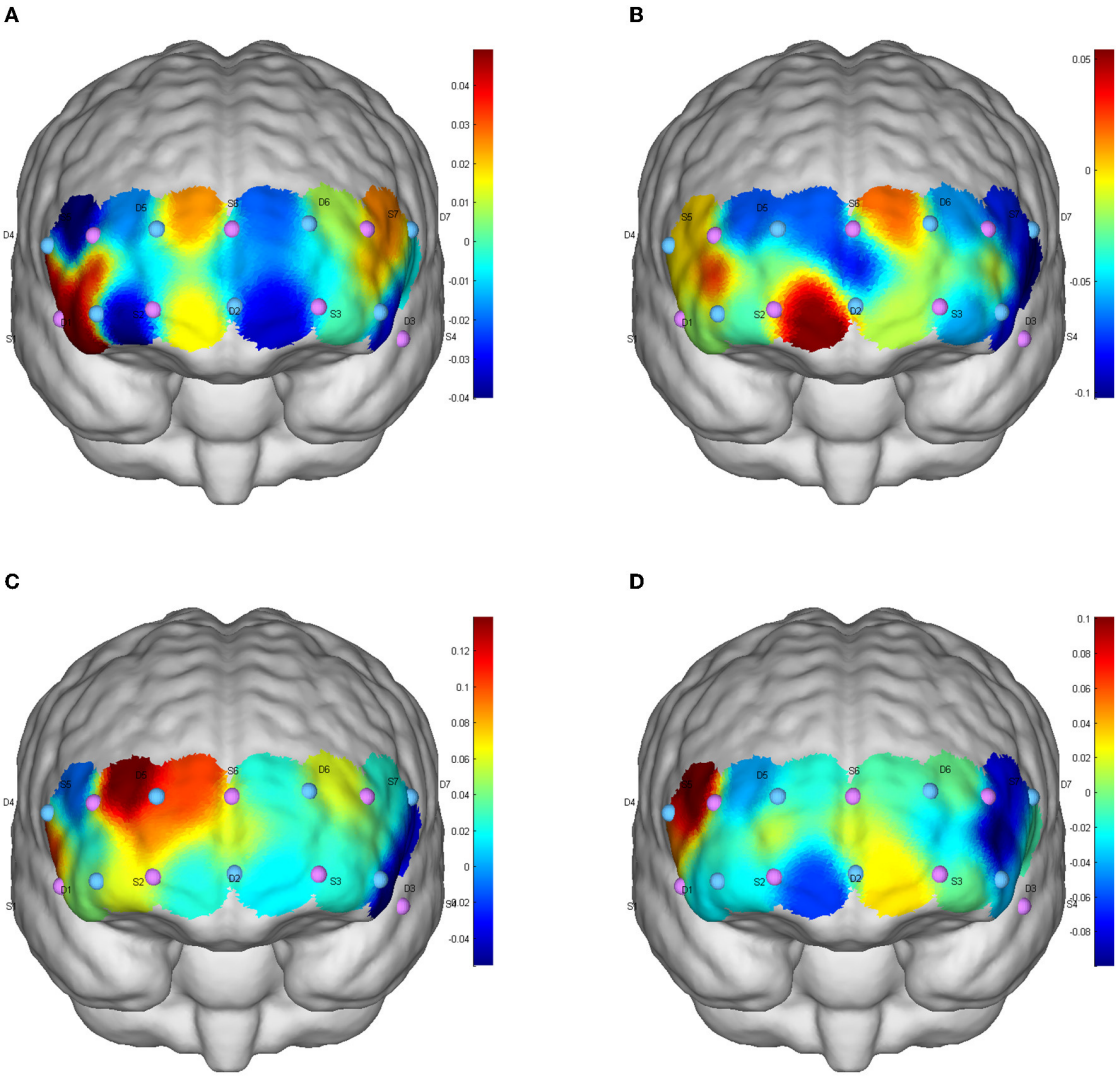
Inhibition function of CBF activation after different exercises in consistent tasks. **(A)** Baseline level; **(B)** aerobic exercise; **(C)** resistance exercise; **(D)** integrated concurrent exercise.

Cerebral blood perfusion in the DLPFC (S5-D5) was significantly increased after resistance exercise compared to baseline under consistent tasks (mean difference = 0.155, *p* = 0.047). Compared with AE, cerebral blood oxygen level in frontal polar region (S6-D5) after RE was significantly increased (mean difference = 0.171, *p* = 0.022); The level of cerebral blood oxygen in DLPFC (S5-D5) was significantly increased after resistance exercise compared with ICE (mean difference = 0.180, *p* = 0.009). Cerebral blood oxygen level in DLPFC (Channels S3-D3, S5-D5, S7-D3) significantly increased after ICE (S3-D3: mean difference = 0.173, *p* = 0.046; S5-D5: mean difference = 0.149, *p* = 0.035; S7-D3: mean difference = 0.171, *p* = 0.025) (Table 3). The brain activation of inconsistent tasks after four tests is shown in Figure 1, and that of consistent tasks is shown in Figure 2.

**Table 3:** Beta mean changes of major brain activation areas in EF test after different exercise.

**EF**	**Task type**	**Brain region**	**Channel**	**Baseline**	**AE**	**RE**	**ICE**
Inhibitory function	Inconsistency	DLPFC	S3-D3	0.011 ± 0.266	0.087 ± 0.196^Δ^	−0.126 ± 0.283	0.047 ± 0.272^Δ^
			S5-D5	0.087 ± 0.160	0.099 ± 0.405	−0.127 ± 0.199	0.021 ± 0.238^Δ^
			S7-D3	−0.050 ± 0.216	0.070 ± 0.363	−0.105 ± 0.210	0.066 ± 0.229^Δ^
		OFC	S2-D1	0.128 ± 0.167	0.130 ± 0.218^Δ^	−0.134 ± 0.251	−0.007 ± 0.275
		IPG	S4-D3	0.008 ± 0.315	0.131 ± 0.262	−0.091 ± 0.341	0.037 ± 0.267
		Broca	S7-D7	0.044 ± 0.139	0.128 ± 0.266	0.006 ± 0.174	0.093 ± 0.197
	Consistent	DLFPC	S5-D5	−0.016 ± 0.192	−0.069 ± 0.362	0.139 ± 0.223^#^	−0.041 ± 0.225^Δ^
		FPA	S6-D5	0.024 ± 0.241	−0.069 ± 0.192	0.102 ± 0.197*	−0.017 ± 0.198
Conversion function		DLPFC	S1-D1	0.071 ± 0.443	0.037 ± 0.429	0.058 ± 0.248	0.071 ± 0.295
			S5-D1	0.100 ± 0.351	0.046 ± 0.360	0.070 ± 0.255	0.118 ± 0.451
			S5-D5	0.077 ± 0.248	0.101 ± 0.341	0.066 ± 0.230	0.046 ± 0.333
			S7-D3	0.040 ± 0.283	0.015 ± 0.415	0.041 ± 0.280	0.152 ± 0.395
			S7-D6	0.095 ± 0.248	−0.063 ± 0.316	−0.014 ± 0.266	0.047 ± 0.329
		FPA	S3-D3	0.111 ± 0.307	0.093 ± 0.327	0.049 ± 0.319	0.133 ± 0.355
			S3-D6	0.082 ± 0.277	0.087 ± 0.281	0.012 ± 0.259	0.090 ± 0.326
			S6-D2	0.065 ± 0.334	0.078 ± 0.299	−0.035 ± 0.212	0.053 ± 0.303
Refresh function		FPA	S2-D5	0.035 ± 0.363	0.056 ± 0.310	0.050 ± 0.295	0.037 ± 0.490
			S3-D6	0.071 ± 0.283	0.118 ± 0.256	0.035 ± 0.367	0.040 ± 0.292
			S6-D5	−0.050 ± 0.276	0.031 ± 0.210	−0.014 ± 0.228	−0.011 ± 0.266
			S6-D6	0.065 ± 0.313	0.123 ± 0.276	−0.021 ± 0.323	−0.009 ± 0.351
		OFC	S2-D1	0.048 ± 0.376	0.182 ± 0.306	−0.015 ± 0.464	0.109 ± 0.384
			S2-D2	0.050 ± 0.374	0.054 ± 0.299	−0.006 ± 0.342	0.028 ± 0.455
			S3-D2	0.058 ± 0.323	0.110 ± 0.287	−0.039 ± 0.313	0.091 ± 0.417
		DLPFC	S7-D6	−0.030 ± 0.292	0.086 ± 0.239	−0.001 ± 0.227	0.091 ± 0.279
		Broca	S5-D4	−0.038 ± 0.267	0.005 ± 0.318	−0.060 ± 0.349	0.052 ± 0.448

AE, aerobic exercise; RE, resistance exercise; ICE, Integrated concurrent exercise.

# *p* < 0.05, indicating a significant difference from the baseline level.

* *p* < 0.05, indicating significant difference with AE. Δ*p* < 0.05, indicating significant difference with RE.

#### 5.3.2. Brain activation during functional conversion tests

**Figure 3:**
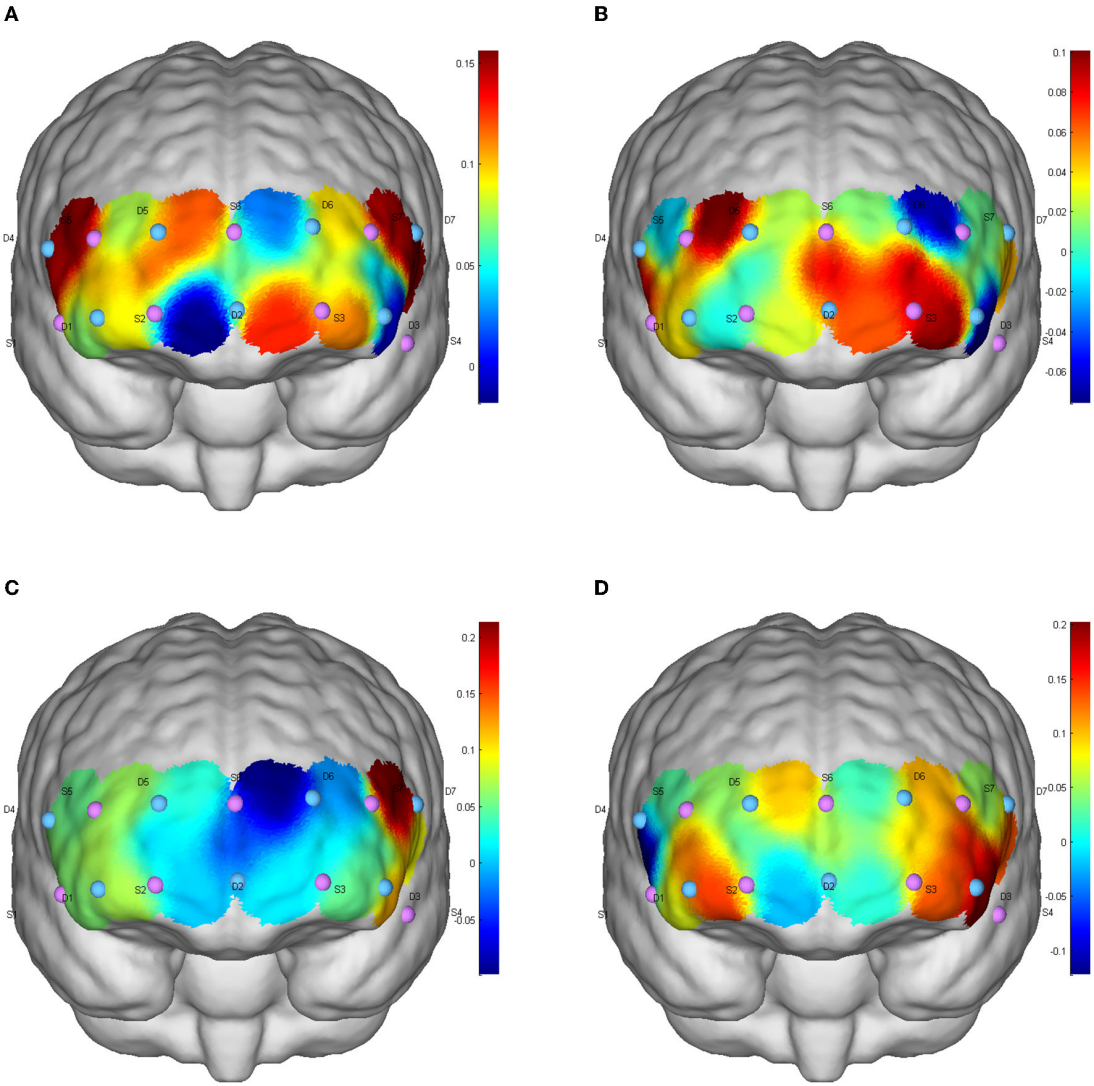
CBF activation after different exercises during the conversion function test. **(A)** Baseline level; **(B)** aerobic exercise; **(C)** resistance exercise; **(D)** integrated concurrent exercise.

There was no significant difference in the level of brain activation after the three exercises, but in the DLPFC (Channels S1-D1, S5-D1, S5-D5, S7-D3, S7-D6) and FPA (S3-D3, S3-D6, S6-D2) showed a trend of increased blood perfusion level. Compared with AE and RE, ICE activated more brain regions and triggered a greater increase in cerebral blood perfusion (Figure 3).

#### 5.3.3. Refresh brain activation during functional test

**Figure 4:**
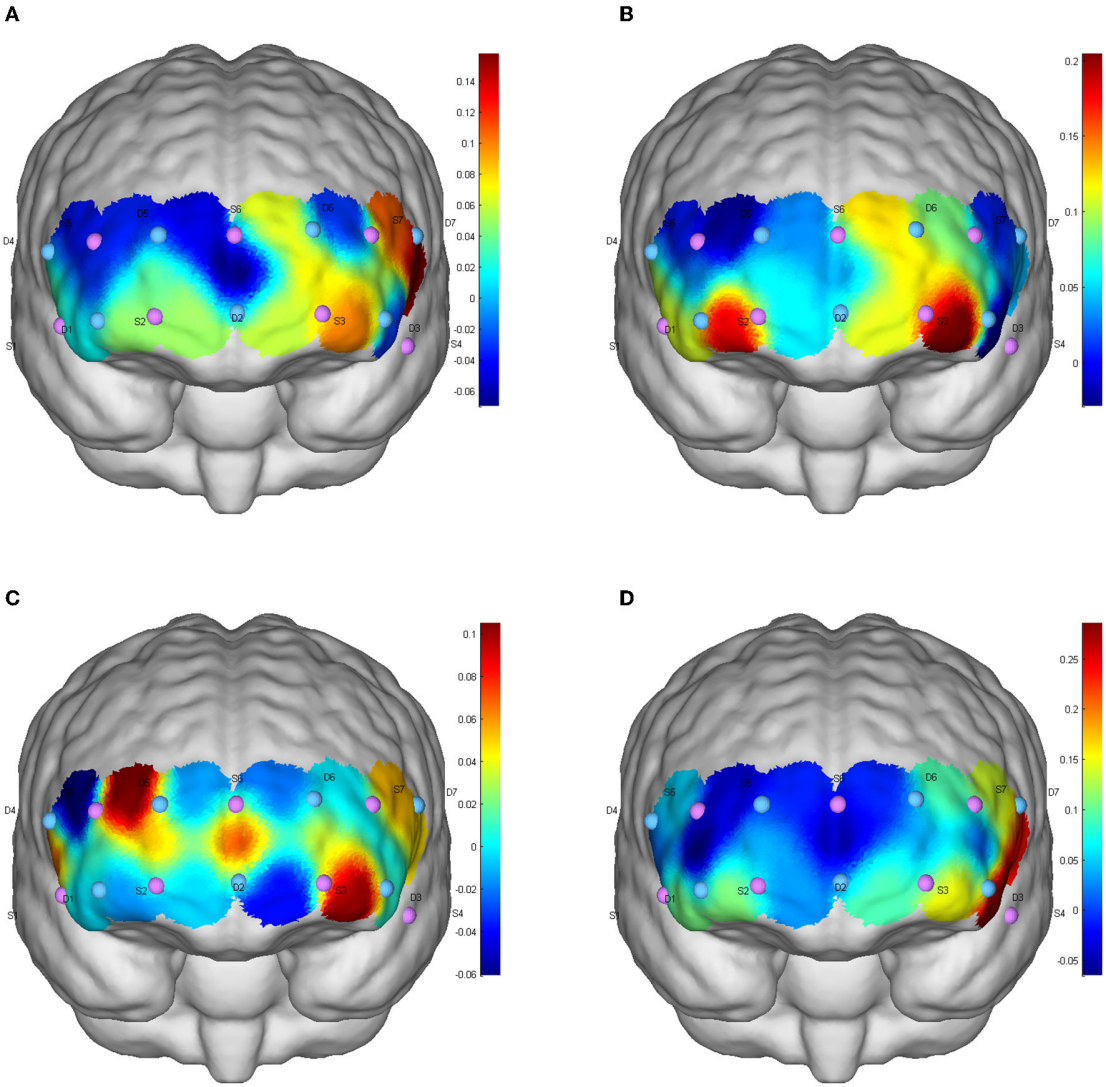
CBF activation after different exercises during the refresh function test. **(A)** Baseline level; **(B)** aerobic exercise; **(C)** resistance exercise; **(D)** integrated concurrent exercise.

There was no significant difference in the level of brain activation after different types of exercise (*p* > 0.05), but FPA brain regions (channels S2-D5, S3-D6, S6-D5, S6-D6), OFC regions (S2-D1, S2D2, S3D2), DLPFC regions (S7-D6) and Pars triangularis Broca's area(Broca; S5-D4) showed increased cerebral blood perfusion, and the brain activation degree of ICE and AE was greater than that of resistance exercise (Figure 4).

### 5.4. Blood glucose data

The results of one-way repeated measure ANOVA showed a significant difference in blood glucose changes (pre-exercise minus post-exercise blood glucose) after different exercise types, *F*_(2,58)_ = 7.542, *p* = 0.003, partial η^2^ = 0.206. Compared with RE, blood glucose decreased significantly after AE (mean difference = −1.25 mmol/L, *p* = 0.005). After exercise, blood glucose decreased from large to small in the order: AE (2.52 mmol/L) > ICE (1.85 mmol/L) > RE (1.28 mmol/L).

## 6. Discussion

### 6.1. Main findings

This study aimed to explore the acute effects of different exercise modes on executive function (inhibition, conversion, and refresh function) in T2DM patients and to reveal the hemodynamic mechanism of relevant brain regions after exercise through the simultaneous collection of cerebral blood oxygenation levels in the prefrontal cortex. This study confirmed that 20 min of moderate-intensity AE, RE, or ICE has different effects on participants' EF and cerebral blood flow activation degree. ICE can synchronize and significantly improve the three executive functions and the corresponding HbO_2_ concentration in brain regions, and RE has limited effects on EF. However, AE only showed a synchronous increase of HbO_2_ concentration in the corresponding brain area of refresh function.

### 6.2. Effect of ICE on executive function

Compared with RE or AE alone, ICE could be more preferred in EF improvements, which is in consistent with previous studies (25). The intermediate effects would exist in metabolism changes. Current studies have found that, compared with exercise focusing on single dimension, multicomponent exercise could significantly improve the overall insulin sensitivity and glucose and lipid metabolism of older adults, and can promote brain blood microcirculation, which would finally improve cognitive function (26, 27). Similar findings were confirmed in older adults (28). This study further revealed that ICE can improve EF and at the same time it can significantly increase blood oxygen level in the DLPFC. In addition, compared with AE, ICE showed more improvements in the conversion function, and synchronously increase the HbO_2_ concentration in the corresponding brain regions. Asynergistic mechanism would be existed in the relations between brain activation degree and EF improvements. It is therefore suggested that clinicians should not limit exercise prescription to single exercise form but encourage multicomponent exercise forms.

### 6.3. Effect of RE on inhibitory function

Results from this study confirmed the safety and efficacy of resistance training in hospitalized T2DM inpatients. Muscle is not only the prominent place of blood glucose metabolism but also one of the essential target tissues of insulin (29). Since numerous studies have confirmed the efficacy of RE in muscle mass and strength, it is reasonably to understand the EF improvements through the increased insulin sensitivity, improved body metabolism, and reduced neuronal damage (30, 31). To date, studies have found that 6 months of progressive RE can improve EF in patients with mild cognitive impairment (32), older women (33), and healthy people (34). In line with previous studies (35–37), this study found that RE showed more efficacy that AE in the improvements of inhibition function. This can be explained by the increased blood flow velocity and blood oxygen level in the prefrontal brain area as well as the degree of nerve activation during RE; all these would provide the necessary basis for the enhanced inhibition function (38, 39). In addition, the performance on resistance movements may involve more inhibitory function. Taking the kicking and hooking movements as an example, they all require the quadriceps muscles of both legs to exert force against the equipment resistance, and lift the legs and maintain the posture for 2–3 s. In the leg lifting and holding process, participants's instinct is to drop their legs because of gravity and equipment resistance. However, such instinct response should be suppressed due to the task requirements, and the leg lifting state should be maintained for a while. The movement process is full of emotional regulation and mood inhibition, which would help explain why RE is more conducive to improve inhibitory function. It is thus would be more preferred for people with diabetes who are short of awareness of diet control and self-management to take RE as daily exercise.

### 6.4. Effect of RE on conversion and refresh function

Results of this study showed that RE can significantly improve three executive sub-functions, but only the consistent task of inhibitory function revealed significant activation of the corresponding brain regions, while the activation level of conversion and refresh functions maintained low. This could be related with participants' physical and mental condition during hospitalization. Most patients have reported physical weakness and lousy mood during hospitalization (40, 41). Negative emotions (e.g., anxiety and tension) can not only increase the speed of CBF but also increase the concentration of HbO_2_ in PFC (42), which may lead to increased cerebral blood perfusion in the baseline test (43), just as showed in Figures 1A). Since hospitalized patients are physically weak, energy cost and HbO_2_ would primarily concentrated in visceral and limb muscles, and thus limiting the increases of HbO_2_ in the brain regions (44, 45).

### 6.5. Effect of AE on refresh function

The positive effects of AE only showed in the rate of correct response to the refresh function test, and the cerebral blood perfusion were increased in the FPA, OFC and Broca region during the test. A synergistic mechanism would exist given the activative level of FPA and OFC and the improvement of refresh function after AE. The Broca region, as the main motor-associated brain region involved in motor imagination, motor execution, and motor behavior (46, 47), shows synchronous activation in exercise. To date, there is no consistent opinion towards the acute effects of AE on brain function. A previous study confirmed that 12 weeks of moderate-intensity AE performed five times a week can improve cognitive function in older adults (48). Wen and his colleagues found that acute AE could increase EF and HbO_2_ concentration in the corresponding brain region of healthy adults (16). Vincent found that acute AE could not significantly improve EF in people with T2DM (49). The variations in the AE effect to middle-aged and older adults with T2DM may be related to the blood glucose fluctuation in patients with T2DM during exercise. The present results revealed decreased blood glucose immediately after AE. The short-term sharp fluctuations in blood glucose may aggravate oxidative stress damage in the hippocampus, which is not conducive to improve cognitive function in patients with T2DM older adults (50, 51). Compared with other exercise forms, AE causes lower arterial CO_2_ concentration, promotes cerebral vascular contraction, and reduces cerebral blood perfusion levels (44, 52). It is recommended that patients with T2DM choose multicomponent exercise forms and closely pay attention to changes of blood glucose level during and after exercise to prevent further damage to EF due to a significant drop in blood glucose. In addition, it is recommended that future studies increase time interval between exercise and cerebral hemodynamic test to obtain more stable data.

This study explored the effects of three kinds of acute exercise on executive function and the mechanism of cerebral hemodynamics, providing a theoretical basis for long-term exercise intervention. This study made a preliminary exploration of the optimal exercise mode for the prevention and treatment of executive function decline in patients with T2DM and increased the scientific nature of exercise in patients with diabetes, which is conducive to the guidance and practice of clinical exercise.

### 6.6. Limitations

Given the limited hospitalization period (5–10 days) and the situation of the COVID-19 pandemic, the time interval within the three exercises (48 h) is relatively shorter than those in the related studies. Future studies are suggested to increase the interval time of exercise intervention to reduce potential effects of exercise fatigue on test outcomes. In addition, the participants in this study were those hospitalized T2DM patients. Although doctors have verified that moderate exercise can be carried out among the participants during such special period, potential interference of other factors could affect outcomes. Future studies are thus suggested to further examine the comprehensive effects of different exercise dosage on EF by reducing participant heterogeneity.

## 7. Conclusion

ICE is preferred for the improvements of EF in T2DM patients, while AE is more conducive to the improvements of refresh function. A synergistic mechanism exists between cognitive function and blood flow activation in brain regions.

## Data availability statement

The original contributions presented in the study are included in the article/supplementary material, further inquiries can be directed to the corresponding author.

## Ethics statement

The studies involving human participants were reviewed and approved by Biomedical Research Ethics Committee, Nanjing Normal University. The patients/participants provided their written informed consent to participate in this study.

## Author contributions

YZ and HW conceived and designed the present study and wrote and critically reviewed the manuscript. HW and WT are responsible for participant recruitment and data acquisition. HW analyzed the data presented in this manuscript. YZ obtained financial support for the present work. All authors approved the final version for submission and were also responsible for all aspects of the study presented in this manuscript.
